# Educational inequalities in multimorbidity at older ages: a multi-generational population-based study

**DOI:** 10.1093/eurpub/ckae096

**Published:** 2024-06-05

**Authors:** Cornelia Wagner, Josephine Jackisch, Natalia Ortega, Arnaud Chiolero, Stéphane Cullati, Cristian Carmeli

**Affiliations:** Population Health Laboratory (#PopHealthLab), University of Fribourg, Fribourg, Switzerland; Swiss School of Public Health (SSPH+), University of Fribourg, Fribourg, Switzerland; Population Health Laboratory (#PopHealthLab), University of Fribourg, Fribourg, Switzerland; Centre for Health Equity Studies, Stockholm University, Stockholm, Sweden; Population Health Laboratory (#PopHealthLab), University of Fribourg, Fribourg, Switzerland; Institute of Primary Health Care (BIHAM), University of Bern, Bern, Switzerland; Population Health Laboratory (#PopHealthLab), University of Fribourg, Fribourg, Switzerland; Swiss School of Public Health (SSPH+), University of Fribourg, Fribourg, Switzerland; Institute of Primary Health Care (BIHAM), University of Bern, Bern, Switzerland; School of Population and Global Health, McGill University, Montreal, Canada; Population Health Laboratory (#PopHealthLab), University of Fribourg, Fribourg, Switzerland; Swiss School of Public Health (SSPH+), University of Fribourg, Fribourg, Switzerland; Quality of Care Service, University Hospitals of Geneva, Geneva, Switzerland; Population Health Laboratory (#PopHealthLab), University of Fribourg, Fribourg, Switzerland; Swiss School of Public Health (SSPH+), University of Fribourg, Fribourg, Switzerland

## Abstract

**Background:**

Social inequalities in multimorbidity may occur due to familial and/or individual factors and may differ between men and women. Using population-based multi-generational data, this study aimed to (1) assess the roles of parental and individual education in the risk of multimorbidity and (2) examine the potential effect modification by sex.

**Methods:**

Data were analysed from 62 060 adults aged 50+ who participated in the Survey of Health, Ageing and Retirement in Europe, comprising 14 European countries. Intergenerational educational trajectories (exposure) were High–High (reference), Low–High, High–Low and Low–Low, corresponding to parental–individual educational attainments. Multimorbidity (outcome) was ascertained between 2013 and 2020 as self-reported occurrence of ≥2 diagnosed chronic conditions. Inequalities were quantified as multimorbidity-free years lost (MFYL) between the ages of 50 and 90 and estimated via differences in the area under the standardized cumulative risk curves. Effect modification by sex was assessed via stratification.

**Results:**

Low individual education was associated with higher multimorbidity risk regardless of parental education. Compared to the High–High trajectory, Low–High was associated with −0.2 MFYL (95% confidence intervals: −0.5 to 0.1), High–Low with 3.0 (2.4–3.5), and Low–Low with 2.6 (2.3–2.9) MFYL. This pattern was observed for both sexes, with a greater magnitude for women. This effect modification was not observed when only diseases diagnosed independently of healthcare-seeking behaviours were examined.

**Conclusions:**

Individual education was the main contributor to intergenerational inequalities in multimorbidity risk among older European adults. These findings support the importance of achieving a high education to mitigate multimorbidity risk.

## Introduction

Multimorbidity—the presence of two chronic conditions or more in an individual—is a growing public health challenge within ageing populations in Western countries as it is associated with poor quality of life, high health care costs and an increased mortality risk.^1^^,^^2^ The prevalence of multimorbidity is higher among adults living in disadvantaged socioeconomic conditions, particularly among those with a low level of education.^3–5^ This educational gradient can be explained by differential access to material and non-material health-beneficial resources.^6^ However, inequalities in multimorbidity may be influenced not only by individual but also by familial factors such as parental education, potentially leading to a long shadow of inequalities.^7^^,^^8^

Parental education may contribute to the amount of cultural and social capital a person has access to, and eventually to social inequalities in offspring health.^9^ Particularly, parental education can affect offspring health via transference of educational attainments, as children of highly educated parents tend to be higher educated themselves. Additionally, parental education can affect offspring health via the promotion of health-beneficial behaviours like preventive healthcare use during the sensitive period of adolescence.^10^ One registry-based study of Danish individuals aged 32–56 years in 2010 reported that both low individual and parental educational levels increased the odds of multimorbidity observed during 8 years of follow-up.^11^ Therefore, how the interplay between individual and parental education might affect the occurrence of multimorbidity in other Western countries and at older ages remains to be examined. What is also unclear is whether educational inequalities in multimorbidity risk differ by sex, as gender-related vulnerability mechanisms could either amplify or diminish the effect of education.^12^ Some sex-specific differences in multimorbidity risk and prevalence have been reported, though findings are inconclusive.^13–15^

Using population-based multi-generational multi-country data, we aimed (1) to assess the role of parental and individual education in shaping educational inequalities in the risk of multimorbidity and (2) to assess potential effect modification by sex. While this is an observational study, we explicitly aimed at estimating causal effects of intergenerational educational trajectories on multimorbidity, drawing on a contemporary approach to causal inference from observational data. Specifically, we built a causal model and defined targeted estimands via counterfactual contrasts.

## Methods

### Data source and study population

Our study population stemmed from the Survey of Health, Ageing and Retirement in Europe (SHARE), a longitudinal cohort study spanning more than 20 European countries.^16–18^ The SHARE study started in 2004 and has been conducted biennially, resulting in a total of eight waves until 2019/2020. Our study's baseline corresponded to wave 5 (2013) being the first wave to include an assessment of parental education. During that survey year, participants were from 15 countries: Austria, Belgium, the Czech Republic, Denmark, Estonia, France, Germany, Israel, Italy, Luxembourg, the Netherlands, Slovenia, Spain, Sweden and Switzerland. In our analysis, we excluded participants from Israel (*n* = 2561) because it is not part of the European continent, and individuals with missing year of birth (*n* = 4). Our target population were individuals in their youth (<20 years) since potential interventions would target educational attainment in that early life period. SHARE measures both parental and individual education retrospectively.

SHARE respondents are a representative sample of all people aged 50 years and older at the time of sampling who have their regular domicile in the respective SHARE country.^19^ Additionally, current partners living in the same household are interviewed at each wave regardless of their age. The response rate at wave 5 (baseline) was approximately 40% resulting in 66 188 participants, including those partners. We excluded participants younger than 50 years (*n* = 1181) at baseline to keep in line with SHARE eligibility rules.

The study population at baseline was composed of 62 442 respondents (Supplementary figure S1). The analytic sample included 62 060 respondents, as we excluded those with a missing date of death (*n* = 90) and missing covariates at baseline (*n* = 292). During follow-up, 11 027 participants became multimorbid (*n* = 24 700 were multimorbid at baseline; 35 727 total), 1303 passed away, and 12 237 were lost during follow-up, resulting in 12 793 participants being non-multimorbid and present at wave 8.

### Causal model, exposure and outcome

**Figure 1:**
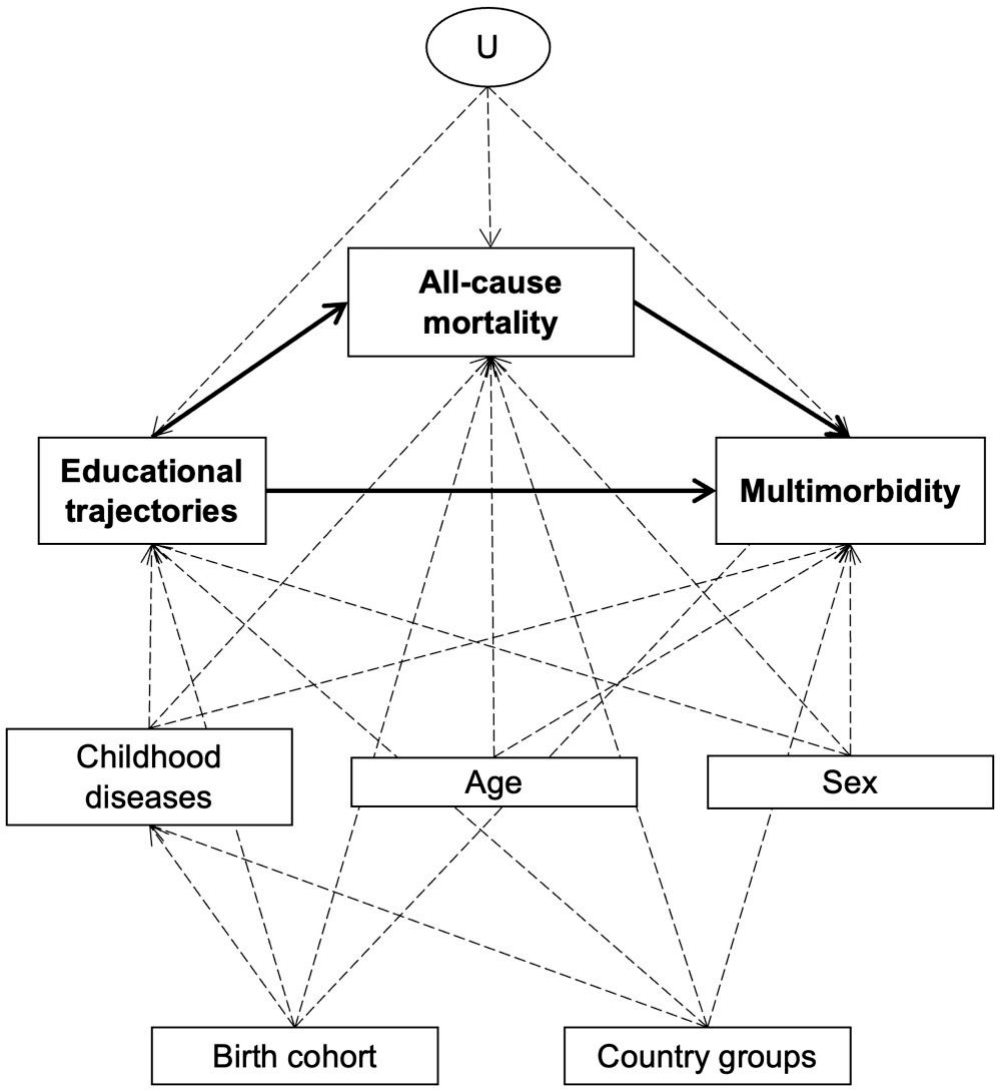
Causal model underlying our study. Solid arrows: putative effect of educational trajectories (exposure) on multimorbidity (outcome) via direct and indirect (all-cause mortality) pathways. Dotted arrows: measured time-invariant confounding factors. U, potential unmeasured confounding.

Our study relied on the causal model reported in figure 1, which focuses on the putative effect of intergenerational educational trajectories (exposure) on multimorbidity (outcome). The total effect of the exposure on the outcome is composed of two pathways, one direct and one mediated by mortality, where deaths are events competing with the occurrence of multimorbidity. Along this pathway, the outcome is prevented when the occurrence of death precedes that of multimorbidity. In other words, death has a deterministic effect on multimorbidity by making it impossible. Four measured potential confounding factors were identified from background knowledge: childhood disease/disability, sex, country groups and birth cohort. Their operationalization is described in Supplementary material.

Educational trajectories were constructed through the combination of individual and parental education, both self-reported by study participants. Parental education was defined as the highest educational attainment reached by either mother or father; in case one was missing, the other’s educational attainment was used. Both individual and parental education were classified as ‘Low’ for any achieved education up to lower secondary level (as per the International Standard Classification of Education (ISCED) 1997, levels 0–2) and as ‘High’ for upper secondary education and beyond (ISCED-1997 level 3 or higher). Using this classification, we obtained four educational trajectories: High–High, Low–High, High–Low and Low–Low, where the first part denotes the parents’ education level and the second part the individual’s education level.

Multimorbidity was operationalized as the self-reported occurrence of minimum two diagnosed chronic conditions from a list of pre-defined conditions. Specifically, participants were asked, ‘Has a doctor ever told you that you had/Do you currently have any of the conditions on this card?’. The list of possible responses spanned 17 different conditions, including ‘other conditions, not yet mentioned’. For this study, we followed a definition of chronic conditions as being permanent in their effects and requiring surveillance, among others.^20^^,^^21^ Thus, we included 13 chronic conditions that met this definition from SHARE’s original list, described in Supplementary material. Conditions such as cataracts, hip fractures and other fractures were excluded.

### Assessment of intergenerational inequalities in multimorbidity

Our research question was formalized by the controlled direct effect (estimand) of educational trajectories on multimorbidity, corresponding to the pathway unmediated by mortality in figure 1. Specifically, we estimated three controlled direct effects by comparing the intergenerational trajectories High–Low, Low–High and Low–Low with the High–High trajectory.^22^^,^^23^ Colloquially, the chosen estimand formalizes educational inequalities in multimorbidity when participants are set or assumed to be immortal, thus blocking the pathway through mortality.^24^ The internal validity of these effect estimates relied on other statistical assumptions described in Supplementary material.

Effects were quantified as multimorbidity-free years lost (MFYL) between ages 50 and 90. MFYL were calculated as differences between educational trajectories in expected years living without multimorbidity between ages 50 and 90. For each level of the exposure, the expected number of years living without multimorbidity was calculated as the area under the corresponding cumulative risk curve standardized by the measured confounders. We chose MFYL to measure the size of inequalities on the absolute scale, to be more relevant in the evaluation of potential policy and public health actions on the examined exposure. In practice, multimorbidity probabilities were estimated via a weighted Kaplan–Meier non-parametric estimator with age as timescale. Since the exact time a participant became multimorbid was unknown, the event was treated as interval-censored between the interview at which multimorbidity was first reported and the last interview the participant reported not being multimorbid. For those multimorbid at study baseline, we considered the interval between age 20 and age at baseline. All participants who did not become multimorbid during follow-up were right censored at the time of wave 8. Death was treated as a censoring event and those participants were included among those lost during follow-up. The Kaplan–Meier derived probabilities were computed using the icenReg R package.^25^ Effect modification (Δ) by sex was implemented by stratifying the data, estimating MFYL for both men and women, and finally calculating the difference in these MFYL.

Weights were the product of two separate stabilized inverse probability weights (IPWs) to account for (1) measured confounding and (2) potential non-random loss during follow-up.^26^^,^^27^ These models’ specification is described in supplementary materials. Confidence intervals (CI) were generated via percentiles of 1000 bootstrap draws with replacement. Within each bootstrapped sample, the effect estimates were the average of 30 multiple imputed datasets for parental or individual education (*n* = 11 295; 18.2%; *n* = 10 503 only for parental education). The imputation model operated under the assumption of missingness at random and its specification is described in supplementary materials.

Ethics approval was not required for this study. We analysed anonymized data and informed consent was obtained at the time of original data collection. All analyses were run in R 4.1.2.

### Sensitivity analyses

To assess the sensitivity of our estimates to the way diseases were ascertained (self-report of diagnosis), we examined the occurrence of only those diseases for which a diagnosis should be independent of healthcare-seeking behaviours. Specifically, we considered as the outcome a self-reported diagnosis of either stroke, cancer (excluding breast, thyroid and prostate cancer) or stomach or duodenal ulcer. The selection of these diseases was informed by expert clinical knowledge of one of the authors (AC). Given the small number of diseases, we focused on the occurrence of these morbidities and not of multimorbidity. Additionally, we repeated this analysis including hypertension and diabetes in the outcome—two diseases for which diagnosis can be related to healthcare-seeking behaviours. We hypothesized that if inequalities were only present when hypertension and diabetes were included, then inequalities or their potential effect modification by sex could be attributed to differences in healthcare-seeking behaviours and not in morbidity occurrence.

To assess the sensitivity of our estimates to the number of co-occurring diseases, we defined multimorbidity as the presence of three or more chronic diseases in an individual (instead of two or more).

To assess the sensitivity of our estimates to the way education was operationalized, we lowered the high-education threshold for parental education. Specifically, we re-classified parental education as ‘Low’ for ISCED-1997 levels 0 and 1 and as ‘High’ for levels 2 or higher. As a further analysis we applied the same operationalization to individual education as well, for those participants born in or before 1927. This analysis was meant to account for the fact that the meaning of a ‘high’ education could have shifted between the parental and individual generations of this study due to the educational expansion taking place in Europe in the middle of the 20th century.^28^ Finally, we assessed the potential bias from the IPW models misspecification by incrementally truncating weights.^27^

## Results

### Analytic sample characteristics

Characteristics of the analytic sample are reported in table 1. Participants had a mean age of 67 years at baseline and 55% were women. Approximately 40% of participants were multimorbid at baseline, and an additional 18% became multimorbid during follow-up (2013–2020). Prevalence of the 13 chronic conditions from which multimorbidity was ascertained is reported in Supplementary table S2. Participants with High–High and Low–Low trajectories accounted altogether for 63% of the non-missing sample, meaning that more than half of the participants attained the same educational level as their parents. Approximately 30% of the participants experienced upward mobility and 6% downward mobility. Additionally, a high education was achieved by nearly eight out of ten participants with high educated parents, and by four out of ten participants with low educated parents. Compared to women, a Low–High trajectory was more prevalent for men (34% versus 28%). By contrast, a Low-Low trajectory was more prevalent among women (41%) compared to men (36%). Finally, men had a higher mortality rate than women.

**Table 1:** Characteristics of analytic sample.

Characteristics	Total	Men	Women
Number of participants	62 060	27 695 (45%)	34 365 (55%)
**Age at baseline** (years), mean and SD	67.1 (±10.0)	67.1 (9.7)	67.1 (10.3)
**Birth cohorts**			
1909–1927	2773 (4.5%)	1036 (3.7%)	1737 (5.1%)
1928–1938	12 312 (19.8%)	5482 (19.8%)	6830 (19.9%)
1939–1945	12 721 (20.5%)	5974 (21.6%)	6747 (19.6%)
1946–1955	22 140 (35.7%)	10 113 (36.5%)	12 027 (35.0%)
1956–1963	12 114 (19.5%)	5090 (18.4%)	7024 (20.4%)
**Childhood disease/disability**			
Yes	18 688 (30.0%)	7731 (27.9%)	10 957 (31.9%)
No	43 372 (70.0%)	19 964 (72.1%)	23 408 (68.1%)
**Multimorbidity** (min. 2 chronic conditions)			
At baseline	24 700 (39.8%)	10 509 (37.9%)	14 191 (41.3%)
During follow-up	11 027 (17.8%)	5054 (18.2%)	5973 (17.4%)
**Limitations with activities of daily living** (min. 1 limitation, at baseline)	7284 (11.7%)	2951 (10.7%)	4333 (12.6%)
**Number of deaths** (2013–2020)	6090 (9.8%)	3259 (11.8%)	2831 (8.2%)
**Death rate**, crude (deaths per 100 000 person-years)	2343	2865	1936
**Educational trajectories**			
High-High	12 589 (20.3%)	5932 (21.4%)	6657 (19.4%)
Low-High	15 607 (25.1%)	7712 (27.8%)	7895 (23.0%)
High-Low	2986 (4.8%)	1034 (3.7%)	1952 (5.7%)
Low-Low	19 583 (31.6%)	8085 (29.2%)	11 498 (33.5%)
Missing	11 295 (18.2%)	4932 (17.8%)	6363 (18.5%)
**Country groups**			
Central and Southern Europe	39 508 (63.7%)	17 947 (64.8%)	21 561 (62.7%)
Eastern Europe	14 054 (22.6%)	5790 (20.9%)	8264 (24.0%)
Scandinavia	8498 (13.7%)	3958 (14.3%)	4540 (13.2%)

SD, standard deviation; Central and Southern Europe, Austria, Germany, Netherlands, France, Switzerland, Belgium, Luxembourg, Spain, Italy; Eastern Europe, Czech Republic, Slovenia, Estonia; Scandinavia, Sweden, Denmark.

### Intergenerational educational inequalities in multimorbidity

**Figure 2:**
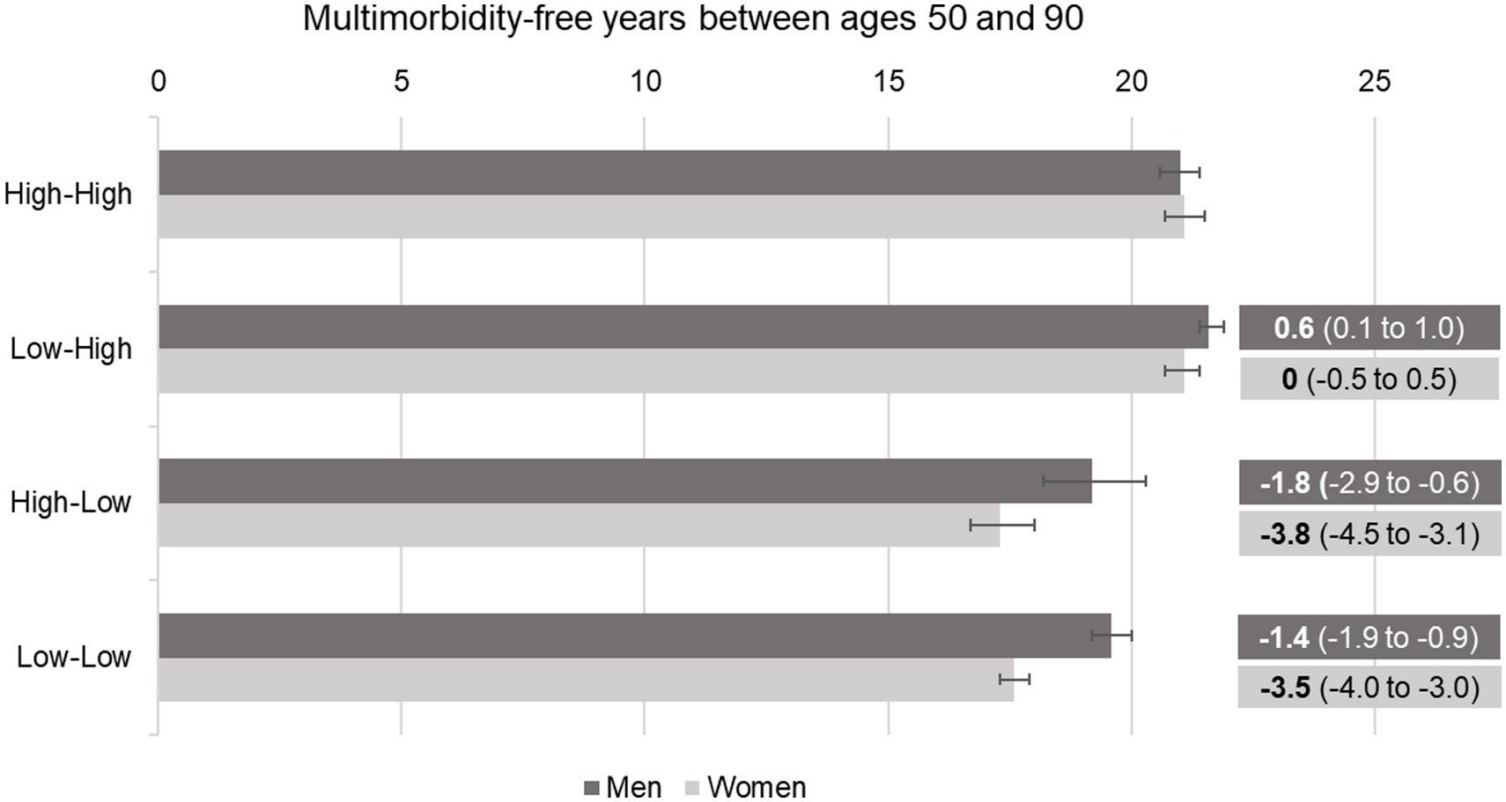
Sex-specific multimorbidity-free years between ages 50 and 90 years and differences in multimorbidity-free years (95% confidence intervals) associated with different educational trajectories compared to the High-High trajectory.

Multimorbidity-free years and MFYL between ages 50 and 90 are reported in table 2. While a High–High trajectory was associated with 21.1 multimorbidity-free years (95% CI: 20.8–21.3), High–Low and Low–Low trajectories were associated with 3.0 (2.4–3.5) and 2.6 (2.3–2.9) fewer multimorbidity-free years, respectively. A Low–High trajectory was associated with 0.2 (−0.1 to 0.5) multimorbidity-free years gained. Taken together, these findings indicate that inequalities in multimorbidity were associated with low individual education regardless of parental education. MFYL were higher for women than for men (table 2 and figure 2). Specifically, inequalities associated with both Low–High and Low–Low trajectories were approximately 2 years longer for women than for men.

**Table 2:** Multimorbidity-free years between ages 50 and 90 years and multimorbidity-free years lost associated with different educational trajectories compared to High–High.

Educational trajectory	**Multimorbidity-free years** (95% CI)	**Multimorbidity-free years lost** (95% CI)
High–High	21.1 (20.8–21.3)	–
Low–High	21.3 (21.1–21.5)	−0.2 (−0.5 to 0.1)
High–Low	18.1 (17.6–18.7)	3.0 (2.4 to 3.5)
Low–Low	18.5 (18.3–18.7)	2.6 (2.3 to 2.9)
**Men**		
High–High	21.0 (20.6–21.4)	–
Low–High	21.6 (21.3–21.8)	−0.6 (−1.0 to −0.1)
High–Low	19.2 (18.1–20.2)	1.8 (0.6 to 2.9)
Low–Low	19.6 (19.2–20.0)	1.4 (0.9 to 1.9)
**Women**		
High–High	21.1 (20.7 to 21.5)	–
Low–High	21.1 (20.7 to 21.4)	0 (−0.5 to 0.5) Δ = 0.6 (−0.1 to 1.3)
High–Low	17.3 (16.7 to 18.0)	3.8 (3.1 to 4.5) Δ = 2.0 (0.6 to 3.2)
Low–Low	17.6 (17.3 to 17.9)	3.5 (3.0 to 4.0) Δ = 2.1 (1.4 to 2.8)

Standardized by sex (in total sample), birth cohort, country group, and childhood disease/disability. Δ represents effect modification.

### Sensitivity analyses

Restricting the outcome to diseases assumed to be independent of healthcare-seeking behaviour yielded the same pattern of magnitude of inequalities across educational trajectories as in the main analysis, although the effect modification by sex vanished (Supplementary table S3). However, including hypertension and diabetes as outcomes reproduced the effect modification by sex observed in the main analysis (Supplementary table S4). This indicates that the sex differences observed in the main analysis could be attributed to differences in healthcare-seeking behaviours and not due to true differences in disease occurrence in men versus women.

When multimorbidity was defined as the presence of three or more diseases (instead of two or more), the pattern and magnitude of inequalities across educational trajectories were similar to those in the main analyses (Supplementary table S5).

The re-classification of parental as well as individual education for participants born in or before 1927 resulted in similar patterns and magnitude of inequalities compared to the one reported in the main results (Supplementary tables S6 and S7). This indicates that the main findings are robust to potential misclassification of education because of potential bias in self-report or because of historical drifts in educational achievements. Lastly, when truncating weights, inequalities were similar to those reported in the main analysis (Supplementary table S8), suggesting negligible bias from the potential misspecification of the IPWs models.

## Discussion

We assessed the educational inequalities in multimorbidity across parent-offspring generations among adults aged 50 and older from 14 European countries. Regardless of parental education, adults with low education experienced a loss of approximately 2.8 years free of multimorbidity compared to those with high education, indicating that these inequalities primarily stem from individual education. Additionally, inequalities were larger for women than for men, although a supplementary analysis indicated that this effect modification could potentially be attributed to differences in diagnosis occurrence and not true disease occurrence.

This is one of few studies to examine inequalities in risk of multimorbidity by intergenerational educational trajectories among older adults. One Danish study reported both individual and parental education to be associated with the risk of certain multimorbidity patterns.^11^ By contrast, our findings from 14 European countries suggest that only individual education contributes to intergenerational inequalities in multimorbidity. However, it is important to highlight that our results point to an indirect effect of parental education whereby parental education affects multimorbidity only via individual education, as participants with highly educated parents were more likely to become highly educated themselves. Additionally, we acknowledge that our study and the Danish study differ in some relevant aspects. Specifically, Schramm et al. used register-based information on 47 chronic conditions, whereas we used self-reported information on 13 different conditions, potentially leading to an underestimation of multimorbidity in our study population. Furthermore, we did not assess inequalities in specific patterns or types of multimorbidity, due to limitations in the available data. Finally, the Danish study estimated odds ratios, which overestimate risk—particularly with a common outcome such as multimorbidity—and suffer from bias related to non-collapsibility. Taken together, the comparability of these two studies is limited.

The observed sex differences in educational inequalities are in line with other studies, whereby women experienced greater health-detrimental effects than men when exposed to low educational attainment.^29^^,^^30^ Ross and Mirowsky propose the theory of resource substitution as an explanation.^30^ This theory states that socioeconomic resources can substitute for each other, meaning the less there is of one resource, the more important other resources become for compensation. The authors suggest that women may have fewer resources than men in society, including power, authority and high earnings, making a high education more important for women. This could partially explain the observed findings, but it is important to not only consider sociological pathways (gender) but also the biological pathways (sex) at play. Research suggests that there are differences in health-relevant biomarkers according to sex at birth, with higher cardiometabolic biomarkers in men and higher inflammatory and neuroendocrine biomarkers in women, and that both sex and gender may lead to these differences.^31^

Sensitivity analyses indicated that the observed effect modification by sex could be due to differences in diagnoses occurrence. Some evidence suggests that women are more likely to visit primary care providers and are thus more likely to be diagnosed with chronic conditions than men.^32^ Ultimately, this is a limitation stemming from how multimorbidity is ascertained in the SHARE dataset. Additional studies with multimorbidity ascertainment of higher validity are required to assess whether there are sex differences in educational inequalities that go beyond self-reported diagnoses.

This study’s findings should be considered within the context of a few potential limitations. The findings may be subject to misclassification bias in the exposure and outcome since they were self-reported. For individual education, the measurement in SHARE was compared to that available in administrative registries and shown to agree in approximately 85% of individuals.^33^ Since most misclassification error stemmed from individuals with low education declaring a high education and appeared to be non-differential,^33^ this misclassification may have led to underestimate the effect of individual education on multimorbidity. For parental education, we could not disambiguate between biological parents and step-parents. Further, the statistical model used for imputing missing educational trajectories, in large part due to missing parental education, operated under the assumption of missingness at random that could have been violated. For the outcome, misclassification could also be due to the operationalization of multimorbidity as diagnosed diseases, meaning undiagnosed diseases are missed, and because the list of diseases was limited. The potential direction of this bias is difficult to determine as it is very likely to be differential and could have possibly masked a direct contribution of parental education to the inequalities. Future studies with more reliable ascertainment of both parental education and multimorbidity are warranted. Additionally, as this is an observational study, we may have bias from unmeasured confounding, and because we were unable to control for finer measured confounding factors due to positivity restrictions.

Further, there could be selection bias as the study population comprises individuals that survived until age 50 or longer. This may have resulted in an underestimation of the inequalities.^34^ For men, this could also explain the observed small reverse inequalities in upwardly mobile individuals compared to High-High. Additional selection bias could arise from nonresponse at baseline and loss during follow-up, although we mitigated the latter by implementing IPWs for follow-up losses. Thus, overall the findings may not be generalizable to the target population. Particularly, it is unclear whether the findings can be applied to more recent birth cohorts, ie those born after 1963. Research suggests that the burden of morbidity—and by extension multimorbidity—is evolving across demographic cohorts. More recent generations, ie those born after 1945, in some European countries experience greater life expectancy, but also an expansion of morbidity.^35^^,^^36^

One key strength of our study is the utilization of a population-based multi-generational and multi-country data sample with multiple multimorbidity assessments. Further, we have adopted a causal framework to estimate marginal inequalities that, contrary to inequalities measured via conditional hazard or odds ratios, are not affected by issues of non-collapsibility and implicit selection bias.^24^

## Conclusion

Our analysis of educational inequalities in multimorbidity risk in later life provides some insights into the intergenerational transmission of social inequalities in health. The findings underscore the role of low individual education as a relevant contributor to higher multimorbidity risk, regardless of parental education. Additionally, inequalities were larger for women than for men, though whether this is a difference in disease diagnoses or in underlying health conditions warrants further investigation. Through a multi-generational, multi-country perspective, this study highlights the importance of achieving high education and of interventions facilitating it, in order to mitigate social inequalities in multimorbidity in later life.

## Supplementary Material

ckae096_Supplementary_Data

## Data Availability

Data are available via registration to the SHARE project website (see www.share-project.org). Key points Multimorbidity risk is higher among older adults with a low achieved education compared to those with high achieved education; growing up in a family with parents of low education, too, may increase the risk of multimorbidity at older ages independently of individual education, but empirical evidence is limited. Additionally, it is not well known whether intergenerational educational gradients in multimorbidity are different for women and men. Low individual education was the main contributor to higher multimorbidity risk, regardless of parental education. Educational inequalities in multimorbidity were approximately twice larger for women than for men, but it is unclear whether this is due to differences in multimorbidity occurrence or due to differences in disease diagnoses. These findings underscore the importance of achieving a high education and of policies facilitating it in order to mitigate multimorbidity risk at older ages.

## References

[1] Dugravot A , Fayosse A, Dumurgier J et al Social inequalities in multimorbidity, frailty, disability, and transitions to mortality: a 24-year follow-up of the Whitehall II cohort study. Lancet Public Health 2020;5:E42–E50. 31837974 10.1016/S2468-2667(19)30226-9 PMC7098476

[2] Marengoni A , Angleman S, Melis R et al Aging with multimorbidity: a systematic review of the literature. Ageing Res Rev 2011;10:430–9. 21402176 10.1016/j.arr.2011.03.003

[3] Pathirana TI , Jackson CA. Socioeconomic status and multimorbidity: a systematic review and meta‐analysis. Australian and New Zealand Journal of Public Health 2018;42:186–94. 29442409 10.1111/1753-6405.12762

[4] Ni Y , Zhou Y, Kivimäki M et al Socioeconomic inequalities in physical, psychological, and cognitive multimorbidity in middle-aged and older adults in 33 countries: a cross-sectional study. Lancet Healthy Longev 2023;4:e618–e28. 37924843 10.1016/S2666-7568(23)00195-2

[5] Wagner C , Carmeli C, Chiolero A, Cullati S. Life course socioeconomic conditions and multimorbidity in old age–A scoping review. Ageing Res Rev 2022;78:101630. 35430301 10.1016/j.arr.2022.101630

[6] Masters RK , Link BG, Phelan JC. Trends in education gradients of ‘preventable’mortality: a test of fundamental cause theory. Soc Sci Med 2015;127:19–28. 25556675 10.1016/j.socscimed.2014.10.023 PMC4420623

[7] Abel T. Cultural capital and social inequality in health. J Epidemiol Community Health 2008;62:e13-e. 18572429 10.1136/jech.2007.066159

[8] Willson AE , Shuey KM, Elder J, Glen H. Cumulative advantage processes as mechanisms of inequality in life course health. Am J Sociol 2007;112:1886–924.

[9] Pishghadam R , Zabihi R. Parental education and social and cultural capital in academic achievement. IJEL 2011;1:50.

[10] Huebener M. The effects of education on health: an intergenerational perspective. J Human Resources 2022;0219-10060R2. 10.3368/jhr.0219-10060R2.

[11] Schramm S , Møller SP, Tolstrup JS, Laursen B. Effects of individual and parental educational levels on multimorbidity classes: a register-based longitudinal study in a Danish population. BMJ Open 2022;12:e053274. 10.1136/bmjopen-2021-053274 PMC8867340 35197340

[12] Bose CE. Intersectionality and global gender inequality. Gender & Society 2012;26:67–72.

[13] Velek P , Luik AI, Brusselle GGO et al Sex-specific patterns and lifetime risk of multimorbidity in the general population: a 23-year prospective cohort study. BMC Med 2022;20:304–11. 36071423 10.1186/s12916-022-02487-x PMC9454172

[14] Marengoni A , Winblad B, Karp A, Fratiglioni L. Prevalence of chronic diseases and multimorbidity among the elderly population in Sweden. Am J Public Health 2008;98:1198–200. 18511722 10.2105/AJPH.2007.121137 PMC2424077

[15] Taylor AW , Price K, Gill TK et al Multimorbidity-not just an older person's issue. Results from an Australian biomedical study. BMC Public Health 2010;10:718–0. 21092218 10.1186/1471-2458-10-718 PMC3001730

[16] Börsch-Supan A , Brandt M, Hunkler C, SHARE Central Coordination Team et al Data resource profile: the Survey of Health, Ageing and Retirement in Europe (SHARE). Int J Epidemiol 2013;42:992–1001. 23778574 10.1093/ije/dyt088 PMC3780997

[17] Börsch-Supan A , Kneip T, Litwin H et al *Ageing in Europe: Supporting Policies for an Inclusive Society*. Berlin: de Gruyter, 2015.

[18] Börsch-Supan A , Malter F. SHARE Wave 5: Innovations & Methodology. Mannheim: MEA, 2015.

[19] Bethmann A , Bergmann M, Scherpenzeel A. *SHARE Sampling Guide–Wave 8*, SHARE Working Paper Series, 33. Munich: SHARE-ERIC, 2019.

[20] Ho IS-S , Azcoaga-Lorenzo A, Akbari A et al Examining variation in the measurement of multimorbidity in research: a systematic review of 566 studies. Lancet Public Health 2021;6:e587–e597. 34166630 10.1016/S2468-2667(21)00107-9

[21] Jungo KT , Cheval B, Sieber S et al Life-course socioeconomic conditions, multimorbidity and polypharmacy in older adults: a retrospective cohort study. PLoS One 2022;17:e0271298. 35917337 10.1371/journal.pone.0271298 PMC9345356

[22] Anker D , Cullati S, Rod NH et al Intergenerational educational trajectories and premature mortality from chronic diseases: a registry population-based study. SSM Popul Health 2022;20:101282. 36353097 10.1016/j.ssmph.2022.101282 PMC9638825

[23] Wagner C , Cullati S, Sieber S et al Intergenerational educational trajectories and inequalities in longevity: a population-based study of adults born before 1965 in 14 European countries. SSM Popul Health 2023;22:101367. 36873264 10.1016/j.ssmph.2023.101367 PMC9974424

[24] Young JG , Stensrud MJ, Tchetgen Tchetgen EJ, Hernán MA. A causal framework for classical statistical estimands in failure‐time settings with competing events. Stat Med 2020;39:1199–236. 31985089 10.1002/sim.8471 PMC7811594

[25] Anderson-Bergman C. icenReg: regression models for interval censored data in R. J Stat Soft 2017;81:1–23.

[26] Westreich D. Epidemiology by Design: A Causal Approach to the Health Sciences. New York, NY, USA: Oxford University Press, 2019.

[27] Cole SR , Hernán MA. Constructing inverse probability weights for marginal structural models. Am J Epidemiol 2008;168:656–64. 18682488 10.1093/aje/kwn164 PMC2732954

[28] Meyer JW , Schofer E. The university in Europe and the world: twentieth century expansion. In: Krücken G, Kosmützky A, Torka M (eds.) Towards a Multiversity?: Universities between Global Trends and National Traditions. Bielefeld: transcript Verlag, 2006: 45–62. 10.1515/9783839404683-003.

[29] Ross CE , Masters RK, Hummer RA. Education and the gender gaps in health and mortality. Demography 2012;49:1157–83. 22886759 10.1007/s13524-012-0130-z PMC3496041

[30] Ross CE , Mirowsky J. Gender and the health benefits of education. Sociol Q 2010;51:1–19. 10.1111/j.1533-8525.2009.01164.x PMC3840544 24288417

[31] Colineaux H , Neufcourt L, Delpierre C et al Explaining biological differences between men and women by gendered mechanisms. Emerg Themes Epidemiol 2023;20:2. 36959612 10.1186/s12982-023-00121-6 PMC10037796

[32] Thompson AE , Anisimowicz Y, Miedema B et al The influence of gender and other patient characteristics on health care-seeking behaviour: a QUALICOPC study. BMC Fam Pract 2016;17:38–7. 27036116 10.1186/s12875-016-0440-0 PMC4815064

[33] Bingley P , Martinello A. Measurement error in the Survey of Health, Ageing and Retirement in Europe: a validation study with administrative data for education level, income and employment. Work Pap Ser 2014;16:2014.

[34] Mayeda ER , Filshtein TJ, Tripodis Y et al Does selective survival before study enrolment attenuate estimated effects of education on rate of cognitive decline in older adults? A simulation approach for quantifying survival bias in life course epidemiology. Int J Epidemiol 2018;47:1507–17. 30010793 10.1093/ije/dyy124 PMC6208270

[35] Jivraj S , Goodman A, Pongiglione B, Ploubidis GB. Living longer but not necessarily healthier: the joint progress of health and mortality in the working-age population of England. Popul Stud (Camb) 2020;74:399–414. 32659174 10.1080/00324728.2020.1767297

[36] Gondek D , Bann D, Ning K et al Post-war (1946-2017) population health change in the United Kingdom: a systematic review. PLoS One 2019;14:e0218991. 31269039 10.1371/journal.pone.0218991 PMC6608959

