# Supplementary material for: Educational inequalities in multimorbidity at older ages: a multi-generational population-based study
Source: Eur J Public Health. 2024 Jun 5;34(4):704–9. doi: 10.1093/eurpub/ckae096 (PMC11293817; doi:10.1093/eurpub/ckae096)
Supplement: ckae096_Supplementary_Data [file ckae096_supplementary_data.pdf]

Supplementary materials

Figure S1 *Flow chart of participants selection.*

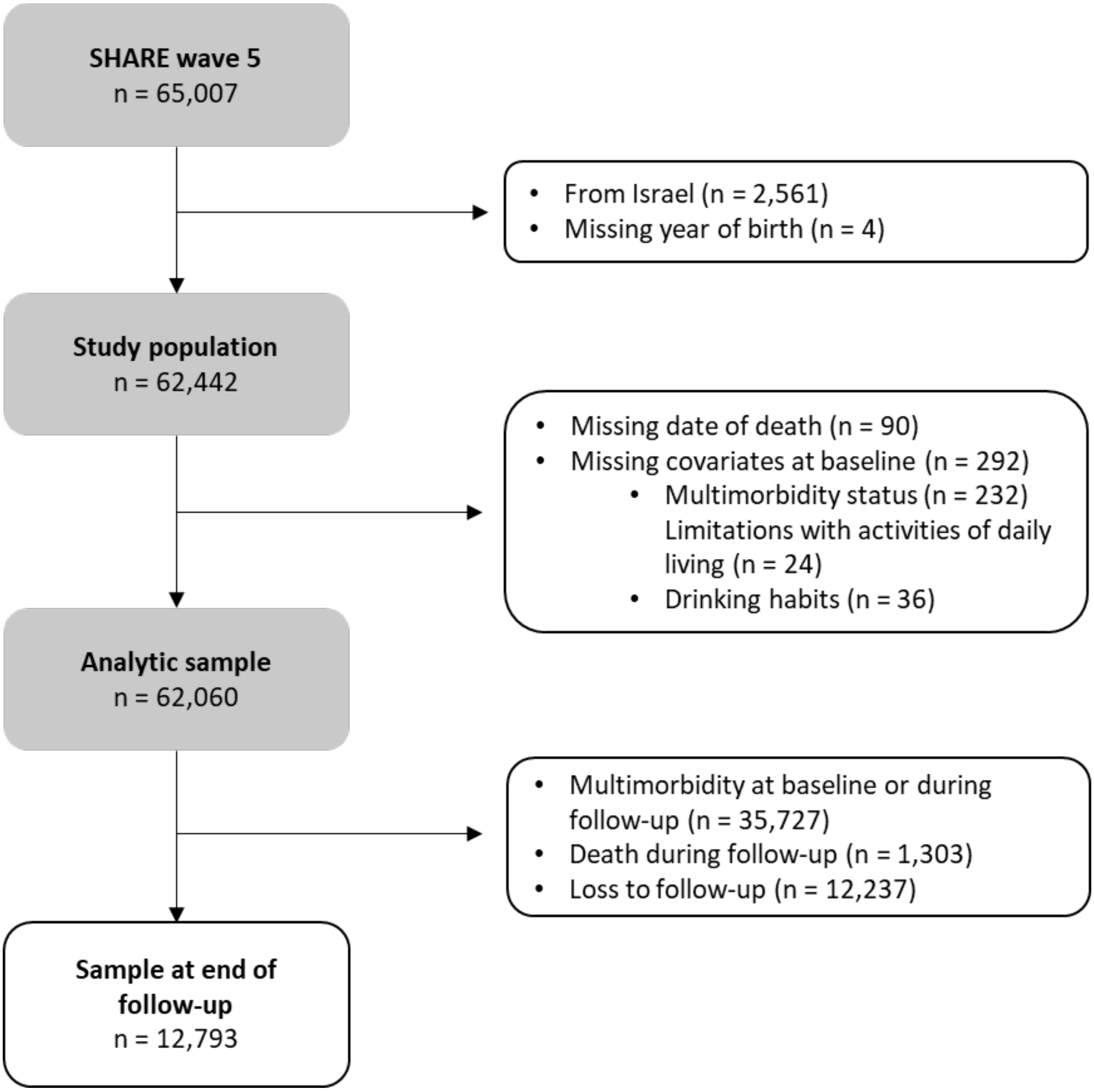

Causal model

The causal model underlying this study was designed to assess the effect of intergenerational educational trajectories (exposure) on multimorbidity (outcome), based on background knowledge. The total effect of the exposure on the outcome is composed of two pathways, one unmediated and one mediated by mortality, with deaths as events competing with the occurrence of multimorbidity. The pathway via all-cause mortality exists because individuals not yet multimorbid who died during follow-up cannot become multimorbid; death therefore has a deterministic effect on multimorbidity by making it impossible. Our inequalities of interest are those corresponding only to the pathway unrelated to mortality. The resulting estimand is the controlled direct effect of educational trajectories on multimorbidity, whereby participants cannot die during follow-up.[1] The assumption of immortal participants is unrealistic but necessary as the other estimand, the total effect, may result in a somewhat different direction and size of inequalities due to the strong effect of education on all-cause mortality.[2]

Mortality data was gathered through end-of-life surveys integrated into the routine SHARE waves. In the case of death of a participant, proxies, such as family members or partners, were invited to provide information regarding the participant's date (month and year) and cause of death. In this study, we considered deaths due to all causes occurring at any point within the follow-up period, i.e. between wave 5 (2013) and wave 8 (2019/20).

The internal validity of the effect estimates relied on the assumptions of positivity, consistency (of the hypothesized interventions on the exposure), no residual confounding, no measurement error of exposure/outcome/confounders, and correct specification of the statistical estimation model.[1, 3] Additionally, since we calculated the direct effect of educational trajectories on multimorbidity, unmediated by death, we relied on the assumption of immortal participants.[1]

Confounders

Measured potential confounders were participant's birth cohort (1909–1927, 1928–1938, 1939–1945, 1946–1955, 1956–1963), sex, country group of residence, and childhood disease/disability. All variables were self-reported and retrieved from the SHARE wave 5 baseline questionnaire. Childhood disease/disability (Yes/No) was defined as either (1) the occurrence of minimum one long-term health condition in childhood and/or (2) a positive response to the question, "Did you ever miss school for a month or more because of a health condition during childhood (that is, from when you were born up to and including age 15)?" (**Table S1**). Countries were categorized in three groups: Scandinavian countries (Sweden, Denmark), Central and Southern European countries (Austria, Germany, Netherlands, France,

Switzerland, Belgium, Luxembourg, Spain, Italy), and Eastern European countries (Czech Republic, Slovenia, Estonia). Countries were combined into groups to reduce the risk of positivity violations and following previous research describing differing intergenerational educational mobility patterns and mortality rates across these groups of countries.[4, 5, 6]

Table S1 *Operationalization of childhood disease/disability. Childhood disease/disability was constructed from either (1) the report of one of the childhood conditions/disability below, or (2) a positive response to the question regarding school absence due to health reasons.*

**"Please look at this card. Did you have any of the diseases on this card during your childhood (that is, from when you were born up to and including age 15)?"**	
1. Asthma<br>2. Respiratory problems other than asthma<br>3. Allergies (other than asthma)<br>4. Meningitis/encephalitis<br>5. Chronic ear problems<br>6. Speech impairment<br>7. Difficulty seeing even with eyeglasses<br>8. Severe headaches or migraines<br>9. Epilepsy, fits or seizures	10. Emotional, nervous, or psychiatric problem<br>11. Childhood diabetes or high blood sugar<br>12. Heart trouble<br>13. Leukaemia or lymphoma<br>14. Cancer or malignant tumour (excluding minor skin cancers)<br>15. Other serious health condition
**"Did you ever miss school for a month or more because of a health condition during childhood (that is, from when you were born up to and including age 15)?"**	

List of chronic conditions considered for multimorbidity

(1) Heart attack including myocardial infarction or coronary thrombosis or any other heart problem including congestive heart failure
(2) High blood pressure or hypertension
(3) High blood cholesterol
(4) Stroke or cerebral vascular disease
(5) Diabetes or high blood sugar
(6) Chronic lung disease such as chronic bronchitis or emphysema
(7) Cancer or malignant tumour, including leukaemia or lymphoma, but excluding minor skin cancers
(8) Stomach or duodenal ulcer, peptic ulcer
(9) Parkinson disease
(10) Alzheimer's disease
(11) Other affective or emotional disorders, including anxiety, nervous or psychiatric problems;
(12) Rheumatoid arthritis
(13) Osteoarthritis, or other rheumatism

Weights and confidence intervals

Weights were the product of two separate stabilized inverse probability weights (IPWs) to account for (1) measured confounding and (2) potential non-random loss during follow-up.[3, 7] The IPW models for confounding included sex (only for the total analytic sample), country group, birth cohort, and childhood disease/disability. The IPW models for follow-up losses included sex (only for the total analytic sample), country group, birth cohort, childhood disease/disability, educational trajectory, and time-varying limitations with activities of daily living (one limitation or more, no limitations).[8] Weight diagnostics encompassing the quality of the weights and the balance of measured confounders across exposure levels were examined. The standardized mean difference for these confounders was <0.01 after IPW, indicating the sample was well-balanced across different educational trajectories. Potential misspecification of the IPW model was ascertained in sensitivity analyses by estimating models using incrementally truncated weights.

Confidence intervals (CI) were generated via percentiles of 1,000 bootstrap draws with replacement. Within each bootstrapped sample, the effect estimates were the average of 30 multiple imputed datasets for parental or individual education (n = 11,295; 18.2%). Our data imputations were carried out through chained equations, operating under the assumption of missingness at random. The prediction variables in the imputation model were sex, country, birth cohort, childhood disease/disability, multimorbidity at baseline, limitations with activities of daily living at baseline, alcohol consumption at baseline, age at baseline, and the cumulative death hazard. Imputations were implemented with the mice R package.[9]

Table S2 *Disease prevalence at baseline (wave 5).*

Disease	Total (n = 62,060)	Men (n = 27,695)	Women (n = 34,365)
Heart attack including myocardial infarction or coronary thrombosis or any other heart problem including congestive heart failure	7,085 (11%)	3,891 (14%)	3,194 (9%)
High blood pressure or hypertension	24,477 (39%)	10,752 (39%)	13,725 (40%)
High blood cholesterol	14,293 (23%)	6,347 (23%)	7,946 (23%)
Stroke or cerebral vascular disease	2,490 (4%)	1,309 (5%)	1,181 (3%)
Diabetes or high blood sugar	7,742 (12%)	3,889 (14%)	3,853 (11%)
Chronic lung disease such as chronic bronchitis or emphysema	3,909 (6%)	1,865 (7%)	2,044 (6%)
Cancer or malignant tumour, including leukaemia or lymphoma, but excluding minor skin cancers	3,542 (6%)	1,600 (6%)	1,942 (6%)
Stomach or duodenal ulcer, peptic ulcer	2,490 (4%)	1,099 (4%)	1,391 (4%)
Parkinson disease	521 (1%)	268 (1%)	253 (1%)
Alzheimer’s disease	1,076 (2%)	438 (2%)	638 (2%)
Other affective or emotional disorders, including anxiety, nervous or psychiatric problems	3,644 (6%)	1,057 (4%)	2,587 (8%)
Rheumatoid arthritis	5,684 (9%)	1,700 (6%)	3,984 (12%)
Osteoarthritis, or other rheumatism	11,691 (19%)	3,750 (14%)	7,941 (23%)

Table S3 *Morbidity defined as min. 1 condition out of 3 conditions less dependent on medical diagnoses (stroke, cancer (excluding breast, thyroid, and prostate cancer), stomach or duodenal ulcer). Morbidity-free years between ages 50 – 90 years and Morbidity-free years lost due to different educational trajectories compared to High-High. Standardized by birth cohort, country group, and childhood disease/disability. Δ represents effect modification.*

**Educational trajectory**	**Morbidity-free years** (95% CI)		**Morbidity-free years lost** (95% CI)	
	**Men**	**Women**	**Men**	**Women**
High-High	**32.5** (32.1 to 32.8)	**33.8** (33.5 to 34.1)	-	-
Low-High	**32.7** (32.4 to 33.0)	**33.6** (33.3 to 33.9)	**-0.2** (-0.7 to 0.2)	**0.2** (-0.2 to 0.6)<br>*Δ = 0.4 (-0.2 to 1.0)*
High-Low	**31.4** (30.4 to 32.3)	**32.4** (31.7 to 33.0)	**1.1** (0.1 to 2.1)	**1.4** (0.7 to 2.1)<br>*Δ = 0.3 (-1.0 to 1.5)*
Low-Low	**31.6** (31.3 to 31.9)	**32.6** (32.4 to 32.9)	**0.9** (0.4 to 1.4)	**1.1** (0.8 to 1.5)<br>*Δ = 0.2 (-0.3 to 0.9)*

Table S4 *Morbidity defined as min. 1 condition out of 5 conditions, three of those less dependent on medical diagnoses (stroke, cancer (excluding breast, thyroid, and prostate cancer), stomach or duodenal ulcer) and two more dependent on medical diagnoses (hypertension, diabetes). Morbidity-free years between ages 50 – 90 years and Morbidity-free years lost due to different educational trajectories compared to High-High. Standardized by birth cohort, country group, and childhood disease/disability. ∆ represents effect modification.*

**Educational trajectory**	**Morbidity-free years** (95% CI)		**Morbidity-free years lost** (95% CI)	
	**Men**	**Women**	**Men**	**Women**
High-High	**17.0** (16.7 to 17.4)	**19.0** (18.6 to 19.4)	-	-
Low-High	**17.9** (17.6 to 18.2)	**19.4** (19.0 to 19.7)	**-0.9** (-1.4 to -0.4)	**-0.4** (-0.9 to 0.1)<br>∆ = *0.5 (-0.3 to 1.2)*
High-Low	**15.8** (14.6 to 16.7)	**15.9** (15.2 to 16.6)	**1.3** (0.3 to 2.4)	**3.1** (2.3 to 3.8)<br>∆ = *1.8 (0.4 to 3.0)*
Low-Low	**16.4** (16.1 to 16.7)	**16.2** (15.9 to 16.4)	**0.6** (0.1 to 1.1)	**2.8** (2.3 to 3.3)<br>∆ = *2.2 (1.5 to 2.9)*

Table S5 *Multimorbidity defined as 3+ chronic conditions. Multimorbidity-free years between ages 50 – 90 years and multimorbidity-free years lost associated with different educational trajectories compared to High-High. Standardized by sex (in total sample), birth cohort, country group, and childhood disease/disability. ∆ represents effect modification.*

**Educational trajectory**	**Multimorbidity-free years** (95% CI)	**Multimorbidity-free years lost** (95% CI)
High-High	**29.3** (29.0 to 29.5)	-
Low-High	**29.2** (29.0 to 29.4)	**0.1** (-0.2 to 0.4)
High-Low	**26.5** (25.9 to 27.0)	**2.8** (2.2 to 3.4)
Low-Low	**26.6** (26.3 to 26.8)	**2.7** (2.4 to 3.0)
**Men**		
High-High	**29.1** (28.7 to 29.5)	-
Low-High	**29.4** (29.1 to 29.6)	**-0.2** (-0.7 to 0.2)
High-Low	**26.8** (25.7 to 27.7)	**2.4** (1.3 to 3.4)
Low-Low	**27.5** (27.2 to 27.8)	**1.6** (1.1 to 2.1)
**Women**		
High-High	**29.3** (29.0 to 29.7)	-
Low-High	**29.0** (28.7 to 29.3)	**0.3** (-0.1 to 0.8)<br>∆ = *0.6 (-0.1 to 1.2)*
High-Low	**26.3** (25.6 to 27.0)	**3.0** (2.3 to 3.8)<br>∆ = *0.6 (-0.7 to 2.0)*
Low-Low	**25.8** (25.5 to 26.1)	**3.6** (3.1 to 4.0)<br>∆ = *1.9 (1.3 to 2.6)*

Table S6 *Educational trajectories with parental education re-classified as "low" (ISCED-1997 0,1) and "high" (ISCED-1997 2+). Multimorbidity-free years between ages 50 – 90 years and multimorbidity-free years lost associated with different educational trajectories compared to High-High. Standardized by sex (in total sample), birth cohort, country group, and childhood disease/disability. ∆ represents effect modification.*

**Educational trajectory**	**Multimorbidity-free years** (95% CI)	**Multimorbidity-free years lost** (95% CI)
High-High	**21.2** (21.0 to 21.4)	-
Low-High	**21.2** (21.0 to 21.5)	**-0.1** (-0.4 to 0.3)
High-Low	**18.5** (18.1 to 18.8)	**2.7** (2.3 to 3.2)
Low-Low	**18.7** (18.4 to 18.9)	**2.5** (2.2 to 2.9)
**Men**		
High-High	**21.0** (20.7 to 21.4)	-
Low-High	**21.8** (21.4 to 22.1)	**-0.7** (-1.2 to -0.2)
High-Low	**18.9** (18.4 to 19.6)	**2.1** (1.4 to 2.7)
Low-Low	**19.9** (19.6 to 20.3)	**1.1** (0.6 to 1.6)
**Women**		
High-High	**21.3** (21.0 to 21.6)	-
Low-High	**20.7** (20.4 to 21.1)	**0.6** (0 to 1.0)<br>*∆ = 1.3 (0.5 to 1.9)*
High-Low	**18.0** (17.6 to 18.5)	**3.3** (2.7 to 3.8)<br>*∆ = 1.2 (0.4 to 2.0)*
Low-Low	**17.6** (17.2 to 17.9)	**3.7** (3.3 to 4.2)<br>*∆ = 2.6 (2.0 to 3.3)*

Table S7 *Educational trajectories with parental education and individual education of those born before or in 1927 re-classified as "low" (ISCED-1997 0,1) and "high" (ISCED-1997 2+). Multimorbidity-free years between ages 50 – 90 years and multimorbidity-free years lost associated with different educational trajectories compared to High-High. Standardized by sex (in total sample), birth cohort, country group, and childhood disease/disability. Δ represents effect modification.*

**Educational trajectory**	**Multimorbidity-free years** (95% CI)	**Multimorbidity-free years lost** (95% CI)
High-High	**21.1** (21.0 to 21.4)	-
Low-High	**21.3** (21.0 to 21.5)	**-0.1** (-0.4 to 0.2)
High-Low	**18.5** (18.1 to 18.9)	**2.6** (2.2 to 3.1)
Low-Low	**18.6** (18.4 to 18.9)	**2.5** (2.2 to 2.9)
**Men**		
High-High	**21.0** (20.7 to 21.3)	-
Low-High	**21.8** (21.4 to 22.1)	**-0.8** (-1.2 to .0.3)
High-Low	**19.1** (18.4 to 19.7)	**2.0** (1.3 to 2.7)
Low-Low	**19.9** (19.5 to 20.3)	**1.1** (0.6 to 1.6)
**Women**		
High-High	**21.2** (20.9 to 21.5)	-
Low-High	**20.8** (20.4 to 21.1)	**0.4** (-0.1 to 0.9)<br>*Δ = 1.2 (0.5 to 1.8)*
High-Low	**18.0** (17.6 to 18.5)	**3.2** (2.6 to 3.7)<br>*Δ = 1.2 (0.3 to 2.0)*
Low-Low	**17.5** (17.2 to 17.9)	**3.7** (3.2 to 4.2)<br>*Δ = 2.6 (1.9 to 3.2)*

Table S8 *Multimorbidity-free years between ages 50 – 90 years and multimorbidity-free years lost due to different educational trajectories compared to High-High when truncating IPWs to the 1st and 99th (Model A), and the 5th and 95th (Model B) percentiles. Standardized by sex, birth cohort, country group, and childhood disease/disability.*

**Educational trajectory**	**Morbidity-free years** (95% CI)		**Morbidity-free years lost** (95% CI)	
	**Model A**	**Model B**	**Model A**	**Model B**
High-High	**21.0** (20.8 to 21.3)	**20.8** (20.6 to 21.1)	-	-
Low-High	**21.3** (21.1 to 21.5)	**21.2** (21.0 to 21.4)	**-0.3** (-0.6 to 0.1)	**-0.4** (-0.7 to 0)
High-Low	**18.1** (17.5 to 18.7)	**18.0** (17.4 to 18.5)	**2.9** (2.3 to 3.5)	**2.8** (2.3 to 3.4)
Low-Low	**18.5** (18.3 to 18.7)	**18.6** (18.3 to 18.8)	**2.5** (2.2 to 2.9)	**2.3** (1.9 to 2.6)

References

1. Young JG, Stensrud MJ, Tchetgen Tchetgen EJ, Hernán MA. A causal framework for classical statistical estimands in failure-time settings with competing events. Statistics in medicine. 2020;39(8):1199-236.
2. Rojas-Saunero LP, Young JG, Didelez V, Ikram MA, Swanson SA. Considering questions before methods in dementia research with competing events and causal goals. American Journal of Epidemiology. 2023:kwad090.
3. Westreich D. Epidemiology by design: a causal approach to the health sciences: Oxford University Press; 2019.
4. Schuck B, Steiber N. Does intergenerational educational mobility shape the well-being of young Europeans? Evidence from the European Social Survey. Social Indicators Research. 2018;139(3):1237-55.
5. Torul O, Oztunali O. Intergenerational educational mobility in europe. Bogazici University, Department of Economics; 2017.
6. Wagner C, Cullati S, Sieber S, Huijts T, Chiolero A, Carmeli C. Intergenerational educational trajectories and inequalities in longevity: A population-based study of adults born before 1965 in 14 European countries. SSM-Population Health. 2023;22:101367.
7. Cole SR, Hernán MA. Constructing inverse probability weights for marginal structural models. American journal of epidemiology. 2008;168(6):656-64.
8. Katz S. Assessing self-maintenance: activities of daily living, mobility, and instrumental activities of daily living. Journal of the American Geriatrics Society. 1983;31(12):721-7.
9. Van Buuren S, Groothuis-Oudshoorn K, Robitzsch A. Package ‘mice’: multivariate imputation by chained equations. CRAN Repos. 2019.
